# Horizontal eyeball akinesia as an initial manifestation of CLIPPERS: Case report and review of literature

**DOI:** 10.1097/MD.0000000000004640

**Published:** 2016-08-26

**Authors:** Xiaohe Hou, Xiaoke Wang, Bo Xie, Weihong Lin, Jun Liu, Dihui Ma, Hong-Liang Zhang

**Affiliations:** a Neuroscience Center, Department of Neurology, the First Hospital of Jilin University; b Department of Neurosurgery, the Second Hospital of Jilin University, Jilin University, Changchun, China.

**Keywords:** Brainstem, CLIPPERS, Corticosteroids, Neuroinflammation, Perivascular infiltration

## Abstract

**Background:**

Chronic lymphocytic inflammation with pontine perivascular enhancement responsive to steroids (CLIPPERS) is a rare chronic inflammatory disorder in the central nervous system (CNS), which is characterized by magnetic resonance imaging (MRI) appearance with punctate and curvilinear gadolinium enhancement “peppering” the pons. Lesions of CLIPPERS mainly involve the pons and the cerebellum. Adjacent structures such as the medulla and the midbrain may also be involved. It is proposed that CLIPPERS is an immune-mediated inflammatory condition characteristic of T-cell-predominant infiltrates and good responsiveness to corticosteroids.

**Methods and Results:**

We report a 46-year-old woman who presented with horizontal eyeball akinesia and gait ataxia with characteristic MRI features of CLIPPERS. The possible pathogenesis, clinical manifestations, imaging features, treatment, and prognosis of this peculiar disorder are summarized.

**Conclusion:**

This report contributes to the clinical understanding of CLIPPERS which may present with horizontal eyeball akinesia as an initial manifestation. The characteristic presentation of a subacute cerebellar and brainstem syndrome and pepper-like gadolinium enhancement was confirmed in this report. Long-term immunosuppressive treatment seems to be mandatory to sustain improvement. Azathioprine alone may be capable of maintaining remission.

## 1 Introduction

Chronic lymphocytic inflammation with pontine perivascular enhancement responsive to steroids (CLIPPERS) was first described in 2010 by Pittock et al^[1]^ as a unique form of brainstem encephalitis centered on the pons. However, the causes and the pathogenesis of CLIPPERS remain poorly understood thus far. The cardinal symptoms at onset include diplopia, gait ataxia, and facial paresthesia. Nystagmus, dysarthria, dysphagia, and other symptoms can also appear when disease progresses. Magnetic resonance imaging (MRI) may disclose characteristic punctate and curvilinear gadolinium enhancement in a “pepper-like appearance” in the pons, midbrain, and cerebellum. Biopsy findings showed perivascular lymphohistocytic infiltrates with a T-lymphocyte predominance. Another major feature of CLIPPERS is clinical and radiological responsiveness to glucocorticosteroids (GCS), and withdrawal of GCS may result in relapses of CLIPPERS.^[1–3]^ Due to the lack of specific biomarkers, it remains a debated issue whether CLIPPERS is an independent new disorder. We herein report a new case and summarize the up-to-date knowledge of the possible pathogenesis, clinical manifestations, and radiological features, as well as treatment modalities and prognosis of CLIPPERS based on 60 previously reported patients with CLIPPERS.

## 2 Case report

**Figure 1:**
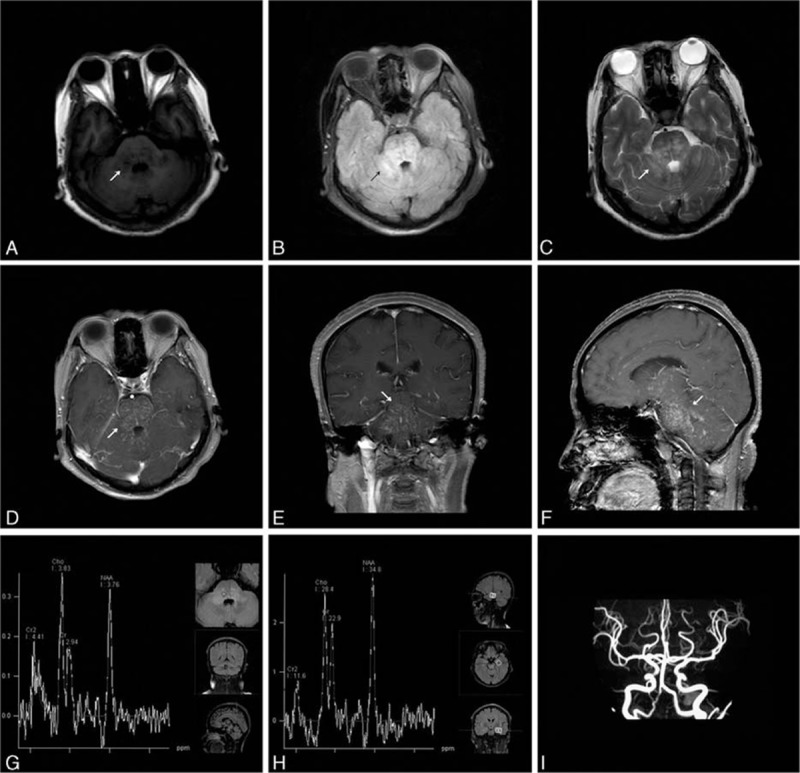
MRI reveals hypointensity on T1 images (A) and hyperintensity on T2 (C) and FLAIR images (B) localized in pons, mesencephalon, and cerebellum, with punctuate gadolinium enhancement in a “pepper-like appearance” centered on the pons in axial (D), coronal (E) and sagittal view (F). MRS (G–H) and MRA examinations (I) are normal. MRA = magnetic resonance angiography, MRI = magnetic resonance imaging, MRS = magnetic resonance spectroscopy.

**Figure 2:**
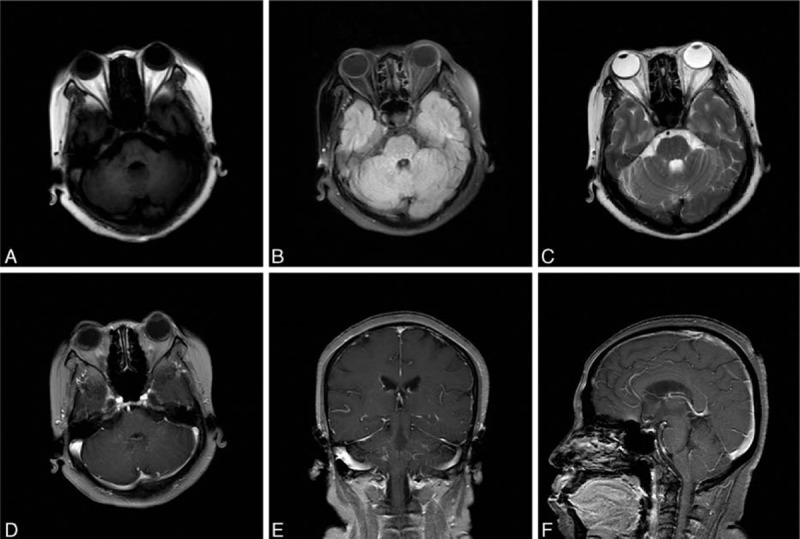
MRI shows resolution of lesions after 4-month immunosuppressive therapy on T1WI (A), T2WI (C), and FLAIR images (B). Axial (D), coronal (E), and sagittal (F) gadolinium-enhanced T1WI show decreased enhancement in the brainstem and the cerebellum. MRI = magnetic resonance imaging.

The reporting of the following case was approved by the ethics committee of the First Hospital of Jilin University, Changchun, China. Though written informed consent was not obtained, the patient's information was anonymized and de-identified. A 46-year-old woman was referred to our hospital with complaints of inability to move her eyeballs horizontally. She also complained of dizziness and gait unsteadiness that progressed over a period of 4 months. Two months after the onset, the patient was treated with dexamethasone (10 mg × 7d, 7.5 mg × 2d, 5 mg × 3d, 2.5 mg × 3d) in the local hospital and gained some improvement in her clinical symptoms. Ten days before admission, her clinical symptoms deteriorated. Upon neurological examination, cerebellar ataxia, dysarthria, decreased memory and calculation ability, left blepharoptosis, inability of eyeball movement in the horizontal direction and loss of positional sense, as well as mild dysmetria on bilateral finger-to-nose, and heel-to-shin testing were noted. Vertical eye movement and ocular convergence were normal. The oculocephalic reflex, corneal reflex, pupillary light reflex, and accommodation reflex were also normal. No nystagmus or facial palsy was observed. No other cranial nerve abnormalities were noted. Limb muscle strength and tendon reflexes in the arms and legs were normal. The Babinski sign and the Chaddock sign were negative bilaterally. Four months after onset, an MRI examination revealed signal abnormalities localized in pons, mesencephalon, and cerebellum on the T1 weighted imaging (T1WI), T2 weighted imaging (T2WI), and fluid attenuated inversion recovery (FLAIR) images with punctuate gadolinium enhancement in a “pepper-like appearance” centered on the pons and without mass effect. The magnetic resonance angiography (MRA) and magnetic resonance spectroscopy (MRS) examinations were normal (Fig. 1). Chest computed tomography (CT) examination was normal. Cerebrospinal fluid (CSF) analysis demonstrated an elevated protein level (0.76 g/L, normal range: 0.15–0.45 g/L) and a normal amount of cells (4 × 10^6^ cells/L; normal range: 0–8 × 10^6^ cells/L). The levels of glucose and chlorides were normal. The permeability of the blood-brain barrier was increased (11.8 × 10^−3^, normal range: <5.0 × 10^−3^). Blood tumor markers were all within the normal range, except for a slightly elevated level of carcino-embryonic antigen (CEA) (5.75 ng/mL, normal range: <5.0 ng/mL). The patient was treated with methylprednisolone (500 mg × 7d, 250 mg × 7d, 120 mg × 7d) followed by tapering dose of oral steroids. Oral steroids treatment was started with 48 mg of methylprednisolone per day and was tapered to 20 mg of methylprednisolone per day, while azathioprine (100 mg/d) was administered as a corticosteroid sparing agent on the third month of the treatment scheme. Oral steroid (20 mg/d) lasted for 45 days and then was tapered and discontinued. We observed marked improvement of the clinical symptoms after the use of methylprednisolone. The symptoms of horizontal eyeball akinesia, dizziness, and gait unsteadiness completely resolved. Memory and calculation ability got much improved. Mild horizontal nystagmus was still notable. MRI showed resolution of gadolinium enhancement lesions after 4-month immunosuppressive therapy (Fig. 2). The patient was still on azathioprine treatment (100 mg/d). She has remained stable with mild impairment of memory and calculation ability but without other symptoms. No more relapses have been observed for 6 months after the treatment.

## 3 Discussion

The diagnostic criteria of CLIPPERS are yet to be determined. The diagnosis of CLIPPERS is made based on the clinical and neuroimaging findings and the exclusion of differential diagnoses. Subacute and progressive brainstem and cerebellar symptoms with characteristic MRI findings showing punctate enhancement in a “pepper-like appearance” centered on the pons are the hallmarks of CLIPPERS syndrome.^[1]^ Our patient's clinical and imaging manifestations were consistent with CLIPPERS. The diagnostic workup was negative for infections, autoimmune disorders, and paraneoplastic syndrome (Table 1). Bickerstaff brainstem encephalitis (BBE) was considered as a differential diagnosis. However, lesions of BBE are usually restricted to the brainstem, with thalamus and cerebellum spared. Besides, pepper-like gadolinium enhancement has not been reported in BBE. More importantly, a relapsing-remitting course of BBE is also rare.^[4]^ The MRS feature of primary central nervous system lymphoma (PCNSL) is a high Cho/NAA ratio. However, PCNSL cannot be completely excluded as patients with PCNSL may have atypical radiological presentations and may respond well to steroids. The differentiation between PCNSL and CLIPPERS is challenging. Taieb et al^[5]^ reported a case that developed a central nervous system B-cell lymphoma 2 years after initial diagnosis of CLIPPERS. They performed biopsy before they made the diagnosis of CLIPPERS. The biopsy in the case showed perivascular CD4-cell infiltrates, which matched with all CLIPPERS findings. However, the patient was ultimately diagnosed as PCNSL. Subacute vascular lesion was excluded by the normal results of MRA. Central pontine myelinolysis was ruled out by the normal serum sodium level and relapsing-remitting course. Autoimmune encephalitis was also ruled out by the clinical and radiological findings, the typical topography of the lesions, and the negative results of the relevant antibodies, that is, anti-NMDA receptor, anti-AMPA1 receptor, anti-AMPA2 receptor, anti-CASPR2, anti-LGI1, and anti-GABA_B_ receptor antibodies in serum and CSF. A chest CT examination helped ruled out sarcoidosis. The diagnosis of multiple sclerosis (MS) was less likely given the lack of typical attacks and MRI lesions. Finally, the diagnosis of CLIIPPERS was established according to the clinical manifestations and typical radiologic findings. Her good response to corticosteroids further supported the diagnosis. Although the neuropathological findings in CLIPPERS have been reported distinct from multiple sclerosis (MS), neuromyelitis optica (NMO), sarcoidosis, neuro-Behçet's disease, glioma, and lymphoma, they are by no means specific.^[6]^ In this case, we did not perform biopsy since it is an invasive procedure and the patient has responded well to corticosteroids. Brain biopsy is necessary when alternative etiologies cannot be ruled out through vigorous investigations, when atypical clinical or neuroimaging findings are noted or when the patients are evidently resistant to GCS.^[6]^

**Table 1:**
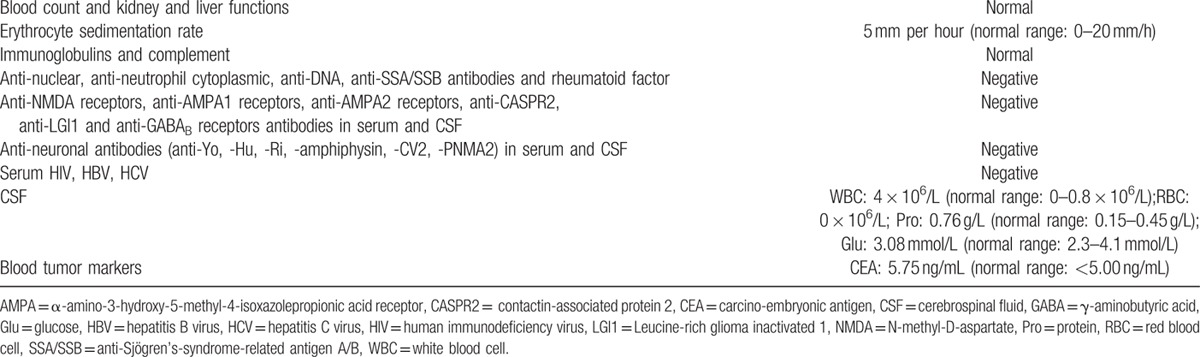
Diagnostic workup.

Characteristic clinical features of CLIPPERS related to involvement of the brainstem, cranial nerves and the cerebellum include gait ataxia, dysarthria, diplopia, and altered facial sensation. Cases may present with cognitive dysfunctions like mnestic deficits and the dys-executive syndrome.^[2,7,6]^ Although cerebellar lesions can cause the cerebellar cognitive affective syndrome which includes impairments in executive, visual-spatial, and linguistic abilities.^[8]^ The cognitive dysfunctions cannot be completely explained by involvement of the cerebellum and the brainstem. Two reported cases with cognitive dysfunctions developed cerebral atrophy over time. Our patient also presented with cognitive deficits including memory loss and calculation ability decline. The cognitive impairments might be due to the lesions in the hippocampus and amygdaloid nucleus. Although MRI showed resolution of gadolinium enhancement lesions after 4-month immunosuppressive treatment without atrophy, more follows-up are necessary. According to the cases which have been reported, the onset age of CLIPPERS ranges from 13 to 86 years. The mean age at onset is 50.2 years. Both sexes are comparably affected. The common symptoms of CLIPPERS are gait ataxia, dysarthria, diplopia, and altered facial sensation in the cases. Eyeball movement disorder and cognitive dysfunctions are relatively rare.

Maintenance treatment is important to prevent further relapses. In this case, azathioprine (100 mg/d) was administered as a corticosteroid sparing agent when methylprednisolone was tapered to 20 mg per day and the patient's condition was stable. Then, we used azathioprine as a monotherapy after oral methylprednisolone was discontinued. This therapy is different from most of the reported strategies. Although corticosteroid sparing agents were proposed to be used to reduce the dose of corticosteroids in long-term therapy in view of their multiple side effects, a majority of the patients used oral corticosteroids alone to maintain remission. Suer et al^[9]^ reported a case who was on azathioprine as maintenance treatment without glucocorticosteroids; follow-up clinical and radiological investigations at the 3rd, 6th, and 12th months were normal. However, some immunosuppressive agents alone appeared incapable of maintaining remission.^[1,6]^

Our case is unique in two aspects. Firstly, her main clinical symptom at onset was inability to move her eyeballs horizontally. Oculomotor abnormalities including gaze palsy, oculomotor palsies, internuclear ophthalmoplegia, and one-and-a-half syndrome are not rare in patients with CLIPPERS. But horizontal eyeball akinesia as an initial manifestation has not been reported. The symptom may relate to involvement of medial longitudinal fasciculus and paramedian pontine reticular formation bilaterally. Secondly, we verified the effect of azathioprine as a monotherapy in maintenance treatment in the case. Most of the cases reported before was treated with oral corticosteroids alone or with additional GCS-sparing agents to maintain remission, because many immunosuppressive agents alone were believed to be incapable of maintaining remission.^[1,6]^ However, chronic glucocorticosteroid therapy is limited by side effects. So, it is important to find some immunosuppressive agents as alternative therapy. More follows-up and evidences are necessary to further prove the effect of azathioprine.

We searched PubMed database until January 1, 2016, for articles published with the search terms “CLIPPERS”. We also reviewed the reference lists of the papers identified by this search. Forty one case studies and a review were included, comprising 60 patients with CLIPPERS. The possible etiology and pathogenesis, clinical characteristics, neuroimaging, diagnostic criteria, and therapeutic management of this disorder are summarized.

The pathogenesis of CLIIPERS remains unknown; no obvious causes are identifiable in most of the patients. Hillesheim et al^[10]^ reported a case of CLIPPERS following influenza vaccination. Ortega et al^[11]^ described a patient with MS who developed CLIPPERS shortly after natalizumab withdrawal. Mashima et al^[12]^ reported a case who developed CLIPPERS after treatment for Hodgkin's lymphoma. Wang et al^[13]^ reported a case of CLIPPERS following herpes zoster infection. To explain the particular features of CLIPPERS, Pittock et al^[1]^ proposed an organ-specific autoimmunity as its possible basis; the target autoantigen of the specific immune-mediated process is likely localized in the perivascular regions. Considering the anatomic arrangement of small intra-axial veins of the central nervous system (CNS), the predominant involvement of brainstem structures might be related to a primary CNS venous inflammatory disorder.^[14]^ Elevated levels of immunoglobulin IgE in serum were noted in several CLIPPERS cases,^[2,15]^ suggesting that immediate and late allergic reactions may contribute to the pathogenesis of CLIPPERS. Interestingly, anti-tuberculous therapy was effective for CLIPPERS.^[16]^ Since rifampicin can inhibit Th17 differentiation and functions, this suggests that CLIPPERS may be a Th17-mediated autoimmune disease.

The cardinal clinical features of CLIPPERS are subacute onset of cerebellar and brainstem syndromes.^[1]^ The onset age of CLIPPERS varies (mean age: 50.2, range: 13–86 years). More men are affected by the disorder than women (Table 2) though the difference is not significant. Symptoms at onset are usually gait ataxia and diplopia.^[1,7]^ Dysarthria, nystagmus, eye movement abnormalities, dizziness, altered facial sensation, pseudobulbar palsy, cognitive deficits, tremor, and other symptoms may appear simultaneously or successively in the course of CLIPPERS.^[1,7,23]^ Tetraparesis and altered sensation of extremities may appear when spinal cord is involved. Tetraparesis may also happen due to lesions in the brainstem which involve the corticospinal tract. According to 60 previously reported cases, most common symptoms of CLIPPERS is ataxia, which was observed in 58 out of 60 cases. Dysarthria (n = 38), diplopia (n = 36), paraparesis/tetraparesis/hemiparesis/paresis of a single extremity (n = 21), nystagmus (n = 20), altered sensation or tingling of the face (n = 16), altered sensation/sensory loss of extremities (n = 16), oculomotor abnormalities (n = 15) are also common characteristics of CLIPPERS (Table 3). Systemic symptoms are generally not a feature of CLIPPERS.^[1]^ The clinical course without specific treatment seems to be relapsing-remitting in nature.^[14]^ MRI may show no abnormalities at the onset of the disease.^[30]^ Serial MRI examinations may be necessary when CLIPPERS is suspected. The hallmark feature of CLIIPERS on MRI is punctate and curvilinear gadolinium enhancement in a “pepper-like appearance” with a perivascular pattern centered on the pons.^[1]^ The gadolinium enhancement may decrease after immunosuppressive therapy. The lesions are typically less numerous and smaller as distance from the pons increases,^[1]^ which may extend into adjacent CNS structures including spinal cord, medulla oblongata, midbrain, cerebellar, corpus callosum, and thalamus. Supratentorial regions may also be involved, such as thalami, capsula interna, basal ganglia, and cerebral white matter, etc.^[7,24]^ Lesions may present as mild to moderate hyperintensitiy on T2 and FLAIR images. Of note is that mass effect does not exclude the diagnosis of CLIPPERS as mass effect might be observed in some patients during relapses.^[14]^ Atrophy of the cerebellum and brachium pontis may appear in the long course of the disease or in severe cases,^[7,35]^ suggestive of neurodegenerative features of the disease.^[21]^

**Table 2:**
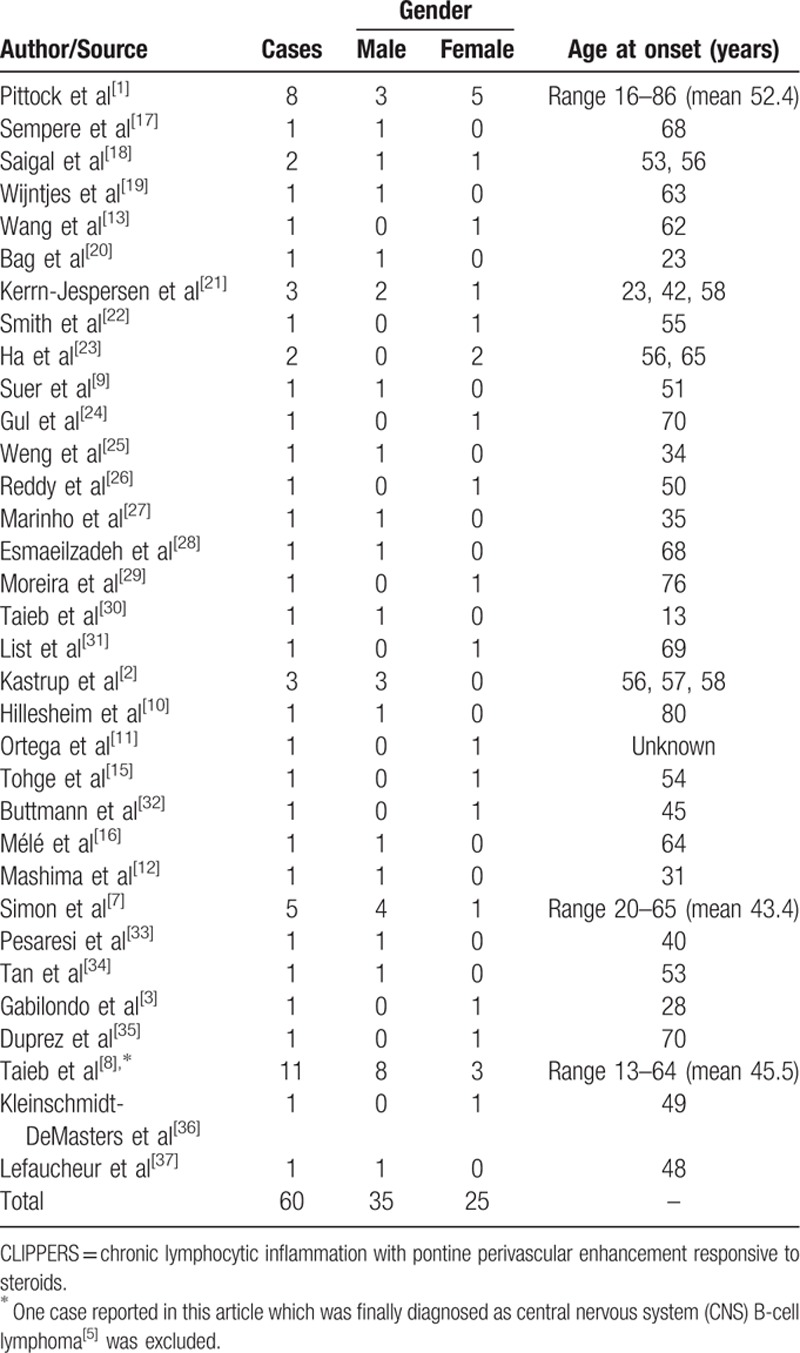
Reported cases of CLIPPERS.

**Table 3:**
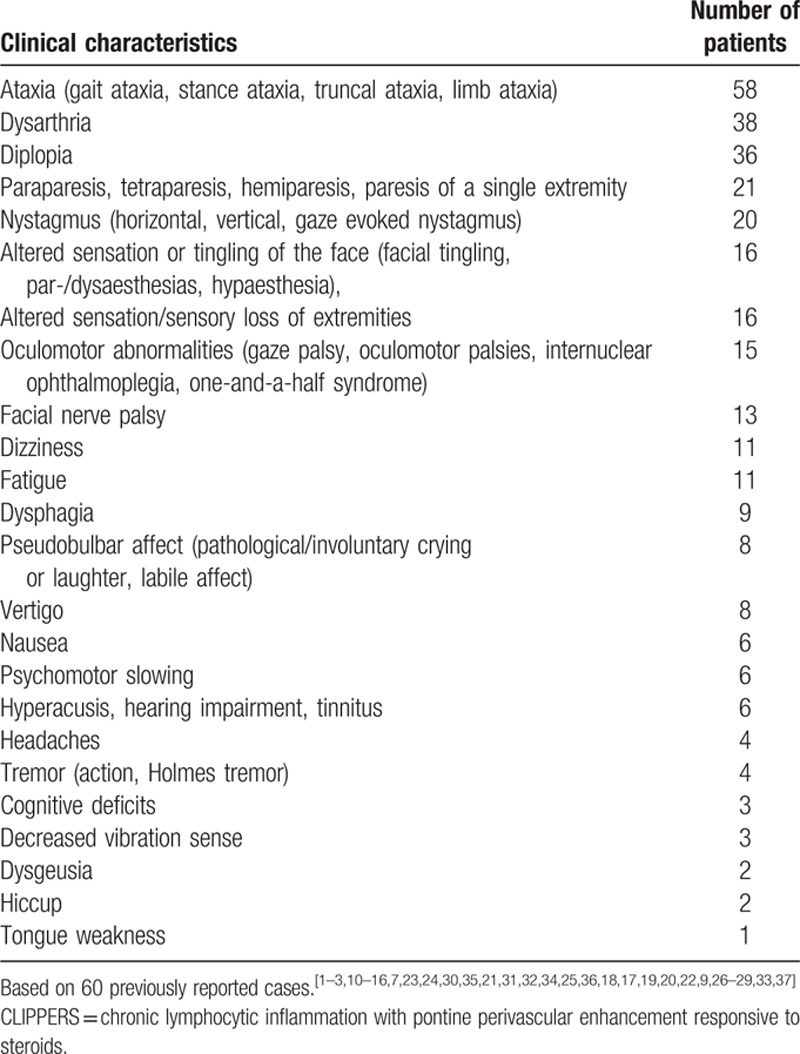
Reported clinical characteristics of CLIPPERS.

There have been no validated diagnostic criteria of CLIPPERS until now. Diagnosis of CLIPPERS is based on clinical, radiological, laboratory and CSF investigations, and brain biopsy when necessary. Other disorders that present with similar lesions should be carefully excluded with extensive investigations. Simon et al^[7]^ highlighted the core clinical, radiological, and histopathological features of the syndrome (Table 4). A diagnostic criteria summarized by Taieb et al^[38]^ include brainstem signs and symptoms, cerebral punctate and curvilinear gadolinium enhancements centered on the pons and cerebellum, good clinical and radiological responsiveness to GCS, absence of evidence of an alternative diagnosis, a relapsing-remitting course fulfilling the criteria above, and perivascular lymphohistocytic infiltrates on brainstem biopsy. Differential diagnoses of CLIPPERS include CNS lymphoma, lymphomatoid granulomatosis, CNS infections, neurosarcoidosis, neuro-Behçet's disease, Sjögren's syndrome, MS, NMO, primary angiitis of the CNS, CNS histiocytosis, acute disseminated encephalomyelitis, Bickerstaff brainstem encephalitis, other inflammatory demyelinating CNS diseases, other autoimmune encephalitides, brainstem tumors, and paraneoplastic disorders.^[1,2,10,11,7,31,39,40,41,32,42,43,6]^

**Table 4:**
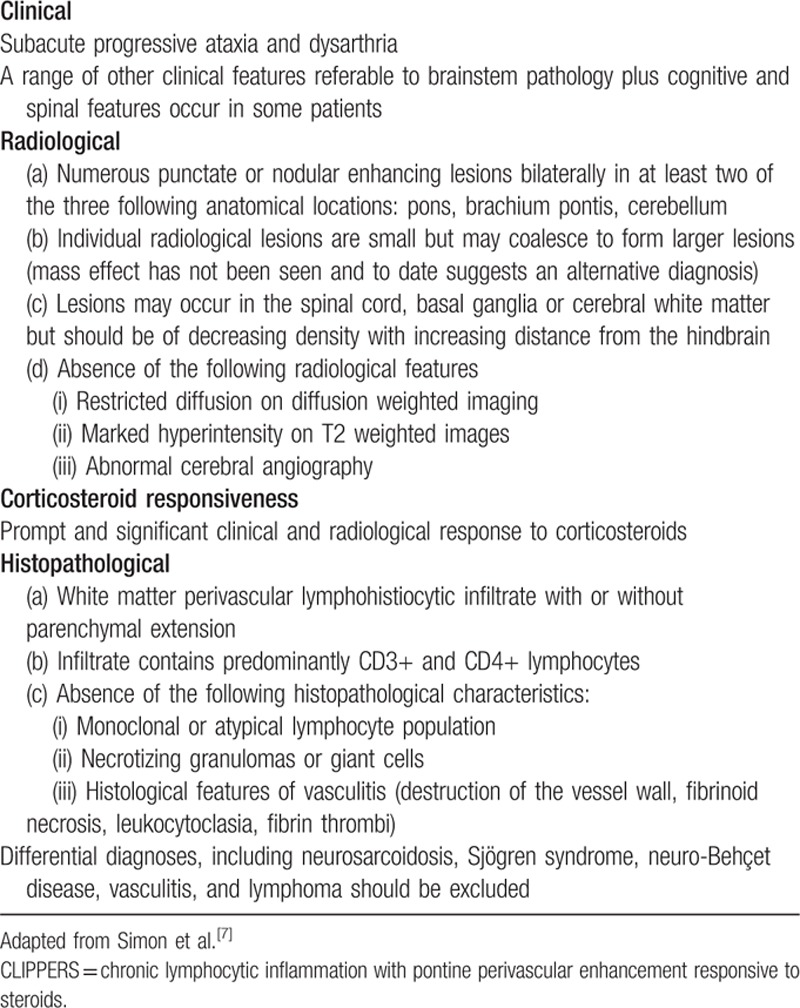
Core features of CLIPPERS.

The unanimous therapy plan is absent due to the relative few cases reported thus far. It is believed that high-dose intravenous methylprednisolone (1 g daily for 5 days) are necessary as soon as possible after a diagnosis has been established so as to prevent progressive clinical worsening.^[14,7]^ Patients usually show early and marked clinical improvement within several days after the use of GCS. Long-term oral corticosteroids (>20 mg/day) seem sufficient to maintain remission and prevent further relapses.^[1,3,14]^ To avoid the side effects of steroids, immunosuppressive agents have been proposed as an add-on choice.^[1,3,14]^ Azathioprine without GCS may be effective to maintain remission.^[9]^ High-dose intravenous methylprednisolone followed by oral GCS should also be started as early as possible in case of relapses. Gabilondo et al^[3]^ tried to taper GCS to 5 mg/day and to use intravenous immunoglobulins (0.4 g/kg/day for 5 days) on a patient, while failed to prevent relapses; cerebellar symptoms reappeared in 2 days after the last dose of intravenous immunoglobulins. It is debatable whether oral hydroxychloroquine is effective. Pittock et al^[1]^ attempted to use oral hydroxychloroquine instead of prednisone to maintain the remission of the symptoms, whereas brain MRI showed radiological progression of the inflammatory lesions. However, Tan et al^[34]^ reported a case who was successfully treated with hydroxychloroquine to induce and maintain remission for 4 years. Mélé et al^[16]^ reported a case with CLIPPERS who was initially misdiagnosed with CNS tuberculosis. The patient was treated with antituberculous therapy including rifampicin, isoniazid, and pyrazinamide and showed clinical and radiologic improvement after the treatment. Combination of levodopa and botulinum toxin injections may be effective for patients of CLIPPERS with tremor.^[23]^ Antiviral therapy is needed when CLIPPERS is accompanied by chronic HBV infection.^[25]^

Taieb et al^[14]^ observed 42 relapses among 12 patients in a mean follow-up period of 5.5 years. On average, 0.63 annual relapses per patient. Recurrence of disease can be provoked by attempts to withdraw or taper GCS below a particularly lower dose limit.^[1–3]^ Long-term GCS treatment seemed necessary for most of the patients with CLIPPERS. Progressive clinical worsening was seen during relapses, which may leave residual neurological sequelae.^[6]^ Inflammatory vessel occlusion was described in biopsy specimens of CLIPPERS^[32,36]^ and stroke mimicking relapses was reported.^[44]^ Saigal and Quencer^[18]^ reported a case of CLIPPERS who had multiple acute lacunar infarcts in basal ganglia. These cases suggest a possible relationship between stroke and CLIPPERS. More follow-up studies are necessary to better delineate the prognosis of CLIPPERS.

In summary, CLIPPERS is an immune-mediated inflammatory disorder of the brainstem, which has been increasingly reported. Irreversible neuronal damage may occur in CLIPPERS.^[35]^ Thus, early usage of high-dose intravenous methylprednisolone is necessary when MRI shows punctate and curvilinear gadolinium enhancement in a “pepper-like appearance” and differential diagnoses are excluded.

## 4 Informed consent

The reporting of the above case was approved by the ethics committee of the First Hospital of Jilin University, Changchun, China. Though written informed consent was not obtained, the patient's information was anonymized and de-identified.
